# The Antagonistic Effect of Glutamine on Zearalenone-Induced Apoptosis via PI3K/Akt Signaling Pathway in IPEC-J2 Cells

**DOI:** 10.3390/toxins13120891

**Published:** 2021-12-12

**Authors:** Tianhu Wang, Jingjing Wang, Tong Zhang, Aixin Gu, Jianping Li, Anshan Shan

**Affiliations:** Institute of Animal Nutrition, Northeast Agricultural University, Harbin 150030, China; wthqh123@163.com (T.W.); jf_jing@163.com (J.W.); neauzt@outlook.com (T.Z.); aixingu@hotmail.com (A.G.)

**Keywords:** zearalenone, glutamine, PI3K/Akt pathway, apoptosis, IPEC-J2 cells

## Abstract

Zearalenone (ZEN) is a non-steroidal estrogen mycotoxin produced by *Fusarium* fungi, which inevitably exists in human and animal food or feed. Previous studies indicated that apoptosis seems to be a key determinant of ZEN-induced toxicity. This experiment aimed to investigate the protective effects of Glutamine (Gln) on ZEN-induced cytotoxicity in IPEC-J2 cells. The experimental results showed that Gln was able to alleviate the decline of cell viability and reduce the production of reactive oxygen species and calcium (Ca^2+^) induced by ZEN. Meanwhile, the mRNA expression of antioxidant enzymes such as glutathione reductase, glutathione peroxidase, and catalase was up-regulated after Gln addition. Subsequently, Gln supplementation resulted in the nuclear fission and Bad-fluorescence distribution of apoptotic cells were weakened, and the mRNA expression and protein expression of pro-apoptotic genes and apoptotic rates were significantly reduced. Moreover, ZEN reduced the phosphorylation Akt, decreased the expression of Bcl-2, and increased the expression of Bax. Gln alleviated the above changes induced by ZEN and the antagonistic effects of Gln were disturbed by PI3K inhibitor (LY294002). To conclude, this study revealed that Gln exhibited significant protective effects on ZEN-induced apoptosis, and this effect may be attributed to the PI3K/Akt signaling pathway.

## 1. Introduction

Zearalenone (ZEN), a mycotoxin produced by *Fusarium*, is one of the vital sources of food contamination. Its ubiquity in food and feed poses a threat to humans and animals health [1]. Studies have shown that after ingesting ZEN-contained foods, the toxic compound was absorbed through the gastrointestinal tract (GIT), metabolized, and distributed to different parts of the body [2]. Reports indicated that ZEN can induce hepatotoxicity, immunotoxicity, hematotoxicity, and genotoxicity, and lead to cell death by inducing oxidative stress, mitochondrial damage, and apoptosis [3,4,5]. Several toxicological models of ZEN’s effects in the body and cells have been carried out in the past years. For instance, previous studies from this lab have shown that ZEN increased the levels of reactive oxygen species (ROS) and repressed the activity and expression of anti-oxidative enzymes in porcine kidney cells (PK15) or porcine intestinal epithelial cells (SIEC02), resulting in cell apoptosis [6,7]. However, little is known and it is worthy to further investigate ways to detoxify for ZEN-poisoned cells and organs.

Glutamine (Gln) is an α-amino acid and the most abundant free amino acid in the body [8]. As a precursor for nucleotide biosynthesis, Gln is one of the crucial substances for intestinal epithelial cell proliferation and integrity repair [9,10]. It was reported that the dietary addition of Gln reduced weaning stress caused intestinal dysfunction by cell proliferation and increased expression of tight junction proteins in weaned pups [11,12]. Similarly, in vitro studies also indicated that Gln could promote the proliferation of intestinal porcine epithelial cell lines [13,14]. In addition, Gln was reported toxic protection effects on the intestinal damage and the intestinal epithelial cell apoptosis caused by clostridium *difficile* toxin-A in the rabbit model [15]. At present, the mechanism of Gln that protects cells from ZEN-induced apoptosis is rarely reported and needs further exploration.

The gastrointestinal tract is a multifunctional and complex organ [16]. It is not only an organ for digestion and absorption of nutrients, but the first barrier to protect animal health from ingested chemicals, food contaminants, and natural toxins. Furthermore, intestinal homeostasis depends on the diverse functions of intestinal epithelial cells [17,18]. Hence, porcine jejunal epithelial cells (IPEC-J2) were selected for the study.

It was hypothesized that Gln might protect the cells against ZEN-induced apoptotic, and it may work via PI3K/Akt signaling pathway. Therefore, the IPEC-J2 cell line was studied as a model to investigate the detoxification of Gln addition on ZEN-induced cells in this study. 

## 2. Results

### 2.1. Effects of ZEN and Gln on Cell Viability

**Figure 1:**
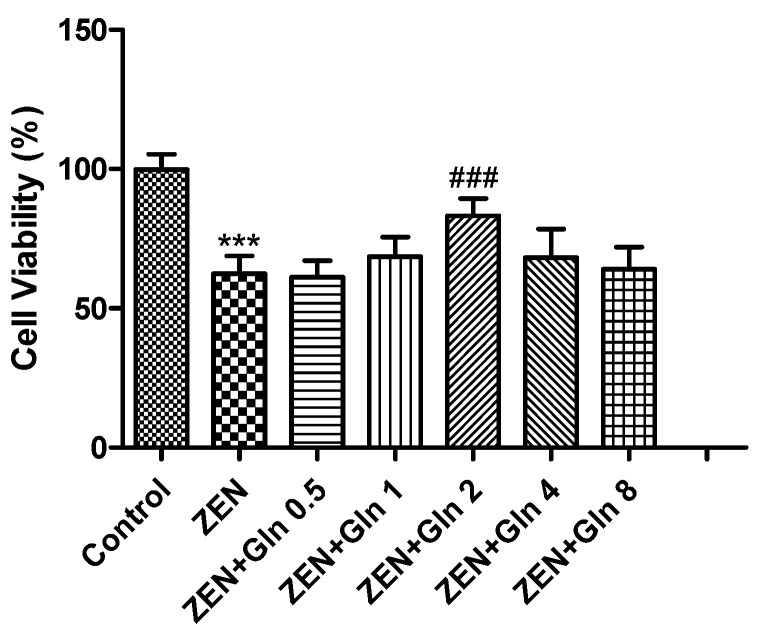
Effects of ZEN and Gln on the viability of the IPEC-J2 cells. Values are expressed as means ± SD of three independent experiments. *** *p* < 0.001 ZEN vs. control. ### *p* < 0.001 ZEN vs. ZEN + Gln 2.

To determine the suitable concentration of Gln in subsequent experiments, the viability of the IPEC-J2 cells (Figure 1) was measured by the CCK-8 method at first. As shown in Figure 1, compared with the control group, exposure to 160 μM ZEN for 48 h, the cell viability was reduced significantly (*p* < 0.001). The addition of 2 mM Gln significantly increased the cell viability compared with the ZEN group (*p* < 0.001).

### 2.2. Effects of ZEN and Gln on the Activities of Enzymes

**Figure 2:**
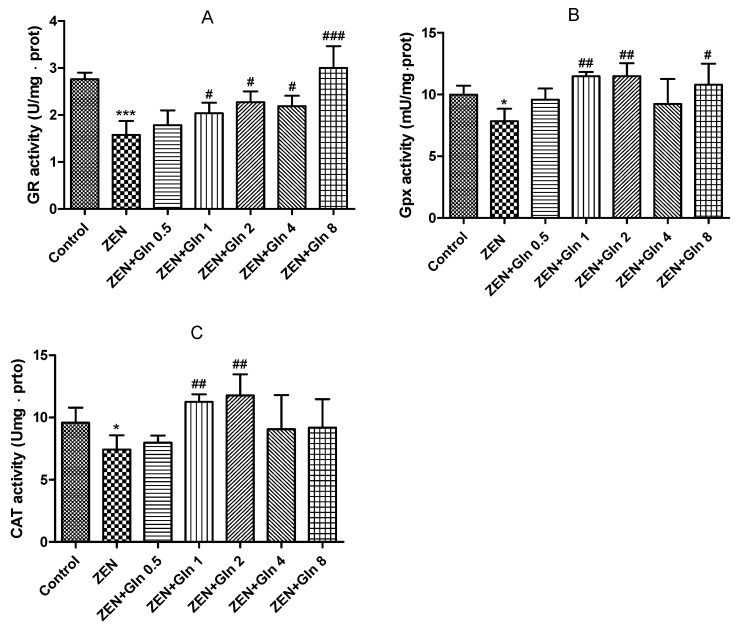
Effects of ZEN and Gln on the activities of the enzymes (GR, Gpx and CAT) in the IPEC-J2 cells. Values are expressed as means ± SD of three independent experiments. * *p* < 0.05 and *** *p* < 0.001 ZEN vs. control. # *p* < 0.05, ## *p* < 0.01, and ### *p* < 0.001 ZEN vs. ZEN + Gln. (**A**) Effects of ZEN and Gln on the activity of GR; (**B**) Effects of ZEN and Gln on the activity of Gpx; (**C**) Effects of ZEN and Gln on the activity of CAT.

When IPEC-J2 cells were exposed to ZEN and different concentrations of Gln for 48 h. As shown in Figure 2, the three enzyme activities (glutathione reductase (GR), glutathione peroxidase (GPx), and catalase (CAT)) decreased significantly upon exposure to ZEN compared with the control group (*p* < 0.05). Compared with the ZEN treatment, no differences were observed in the three enzyme activities at 0.5 mM Gln; however, the level of 1 and 2 mM Gln were showed significant increases (*p* < 0.05) in these three kinds of enzymes. Meanwhile, the concentration of 4 and 8 just observed improvements in one kind of enzyme (Gpx and CAT), respectively. Based on these data, a protective concentration of Gln (2 mM) was selected and incubated with ZEN for 48 h in subsequent experiments.

### 2.3. Intracellular ROS Generation

**Figure 3:**
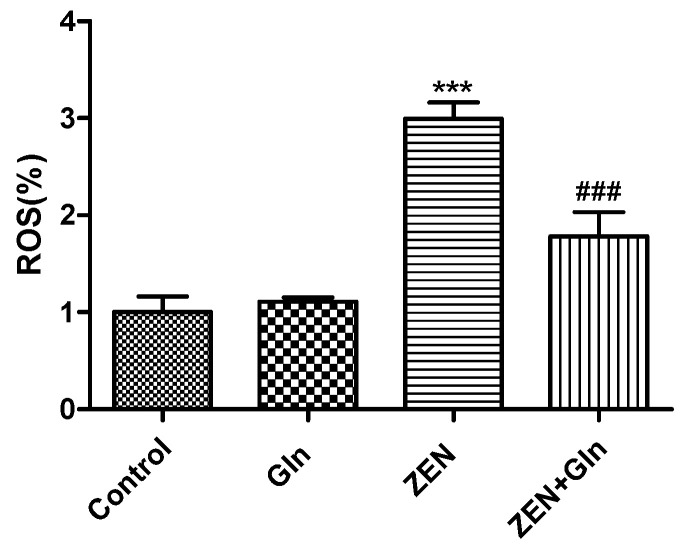
Effects of ZEN and Gln on intracellular ROS production. Values are expressed as means ± SD of three independent experiments. *** *p* < 0.001 ZEN vs. control. ### *p* < 0.001 ZEN vs. ZEN + Gln.

To determine changes in oxidative damage, IPEC-J2 cells were exposed to different drugs for 48 h. The ROS production results were obtained by the fluorescein assay (Figure 3). The figure showed that the level of ROS was significantly higher in the ZEN group than that in the control group (*p* < 0.001). Compared with the ZEN group, the intracellular ROS production was decreased significantly after Gln addition (*p* < 0.001).

### 2.4. Intracellular Ca^2+^

**Figure 4:**
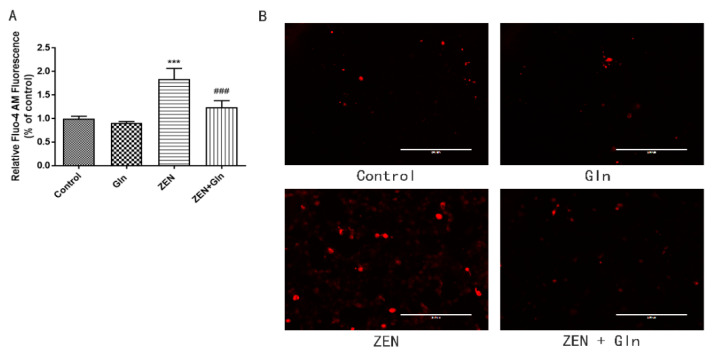
Effects of ZEN and Gln on intracellular Ca^2+^ production. (**A**) Values are expressed as means ± SD of three independent experiments. (**B**) Fluorescence microscopy observation of intracellular Ca^2+^ fluorescence intensity, Scale bar: 200 µm. *** *p* < 0.001 ZEN vs. control. ### *p* < 0.001 ZEN vs. ZEN + Gln.

IPEC-J2 cells were incubated with Fluo-4 AM, bound to Ca^2+^ to produce strong fluorescence. As shown in Figure 4, compared with the control group, ZEN-induced levels of intracellular Ca^2+^ increased significantly (*p* < 0.001). Gln supplementation significantly reduced intracellular levels of Ca^2+^ in the ZEN-induced cells (*p* < 0.001). 

### 2.5. Immunofluorescence Staining of Cells

**Figure 5:**
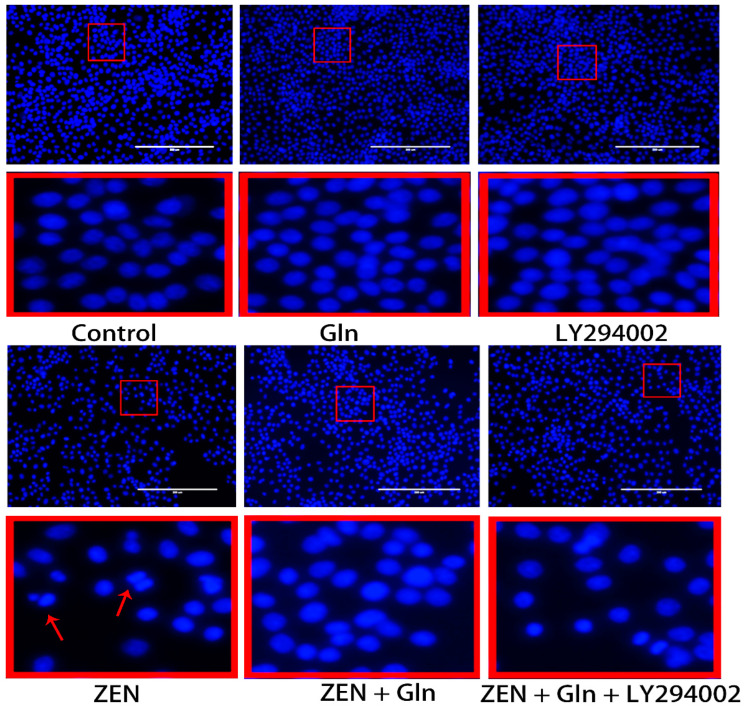
Effects of ZEN, Gln, and LY294002 on the apoptotic nuclei (Hoechst 33258 staining). The red color frame in the figure indicates the obvious change area. The arrow represents a change in nuclear morphology. Scale bar: 200 µm.

The morphologic changes of apoptotic nuclei were observed by fluorescence microscopy with Hoechst-33258 staining (Figure 5). In control group cells, the nuclei displayed uniformly blue-stained with a smooth appearance. However, uneven nuclear staining, nuclear condensation, and fragmentation of nuclei were shown clearly in the ZEN group. In Comparison with the ZEN group, although Gln addition reduced nuclear shrinkage and rupture, pretreatment with LY294002 that did not reduce nuclear apoptosis.

### 2.6. Apoptosis Rate in IPEC-J2 Cells 

**Figure 6:**
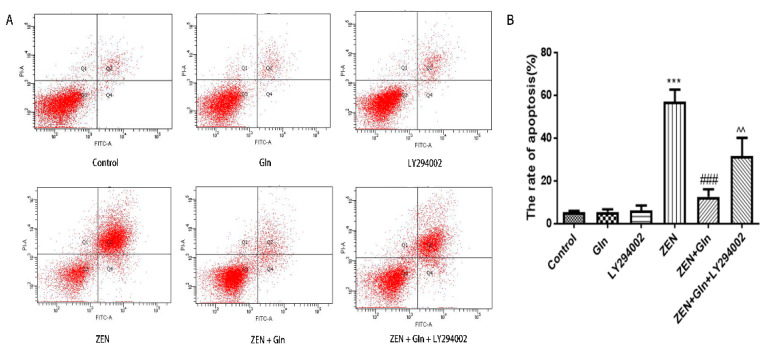
(**A**) The apoptotic cells were determined by annexin V-FITC/PI staining using flow cytometry. The Q1, Q2, Q3, and Q4, respectively, represented dead cells, the late cells apoptosis, normal cells, and the early cells apoptosis. Apoptosis was the sum of early apoptosis and late apoptosis. (**B**) The percentage of IPEC-J2 cells apoptosis was shown in statistical analysis. Each value represents the mean ± SD of the three independent experiments. *** *p* < 0.001 ZEN vs. control, ### *p* < 0.001 ZEN vs. ZEN + Gln. ^^ *p* < 0.01 ZEN + Gln vs. ZEN + Gln + LY294002.

The Annexin V/FITC/PI apoptosis kit was used to analyze different drugs effects on apoptosis of IPEC-J2 cells. As shown in Figure 6A, ZEN induced a significant increase in the number of early apoptotic cells (Q2), as well as in the number of late apoptotic cells (Q4) in IPEC-J2 cells. The total apoptotic cell proportion was increased by 56.8% (Figure 6B) compared with the control group. Compared with the ZEN group, Gln addition significantly reduced early apoptosis and late apoptosis, and the total apoptotic cell proportion (12.5%) was decreased by 44.3%. In addition, pretreatment with LY294002 significantly increased late apoptosis, and the total cell apoptotic rate (31.6%) was increased by 19.1% compared with the ZEN + Gln group. This result was consistent with the results of nuclear apoptosis staining.

### 2.7. The mRNA Expression of Apoptosis-Related Genes

**Figure 7:**
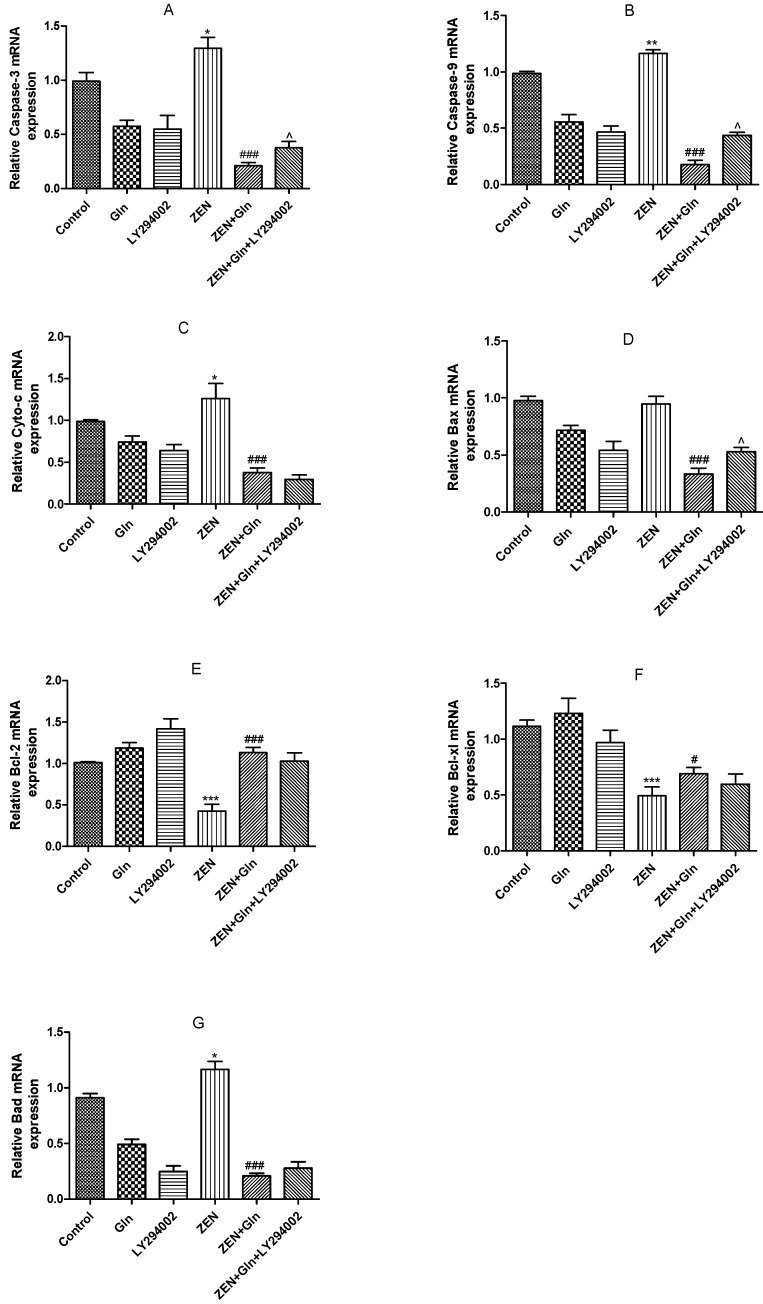
Effects of ZEN, Gln and LY294002 on the apoptosis-related genes in the IPEC-J2 cells. Values are expressed as means ± SD of three independent experiments. * *p* < 0.05, ** *p* < 0.01, and *** *p* < 0.001 ZEN vs. control. # *p* < 0.05 and ### *p* < 0.001 ZEN vs. ZEN + Gln. ^ *p* < 0.05 ZEN + Gln vs. ZEN + Gln + LY294002. (**A**) Effects of ZEN, Gln and LY294002 on the mRNA expression of Caspase-3; (**B**) Effects of ZEN, Gln and LY294002 on the mRNA expression of Caspase-9; (**C**) Effects of ZEN, Gln and LY294002 on the mRNA expression of Cyto-c; (**D**) Effects of ZEN, Gln and LY294002 on the mRNA expression of Bax; (**E**) Effects of ZEN, Gln and LY294002 on the mRNA expression of Bcl-2; (**F**) Effects of ZEN, Gln and LY294002 on the mRNA expression of Bcl-xl; (**G**) Effects of ZEN, Gln and LY294002 on the mRNA expression of Bad.

To further investigate the effects of these drugs on cell apoptotic, the mRNA expression of apoptosis-related genes was measured. As shown in Figure 7, compared with the control group, ZEN induced a significant increase in the mRNA expression levels of pro-apoptotic genes: Caspase-3, Caspase-9, Cytochrome c (Cyto-c), and Bad (*p* < 0.05). Conversely, the mRNA expression of anti-apoptosis genes (Bcl-xl and Bcl-2) was significantly reduced (*p* < 0.001). After Gln addition, the mRNA expression of five pro-apoptotic genes (Caspase-3, Caspase-9, Cyto-c, Bax and Bad) were significantly down-regulated (*p* < 0.001), anti-apoptotic genes (Bcl-xl and Bcl-2) were significantly up-regulated (*p* < 0.05). Compared with the ZEN + Gln group, pretreatment with LY294002, the mRNA expression of three pro-apoptotic genes (Caspase-3, Caspase-9, and Bax) were increased significantly (*p* < 0.05) and had no significant effect on anti-apoptotic genes.

### 2.8. Immunofluorescence

**Figure 8:**
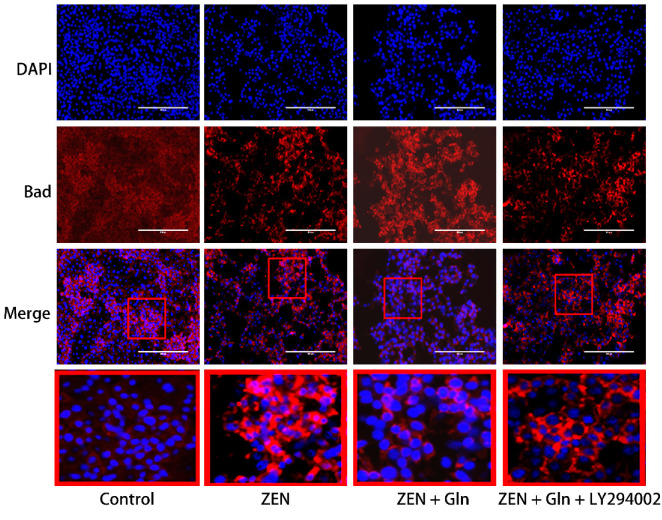
Expression of Bad in IPEC-J2 cells. Cells were stained with antibodies for Bad and detected by immunofluorescence after treatment. The images were collected by the nuclei showed blue fluorescence after counterstaining with DAPI. The red color frame in the figure indicates the obvious change area. Scale bar: 200 µm.

The Bad protein is involved in initiating apoptosis. Next, we investigated the expression of Bad by immunofluorescence. As displayed in Figure 8, in the control group, the fluorescence of Bad was very weak. While in the ZEN group, Bad fluorescence expression was strongly positive, distributed around the nucleus in a spotted manner and decreased number of IPEC-J2 cells. In the Gln + ZEN group, the immunostaining of Bad was relatively weaker than the ZEN group, and there was no distribution pattern of aggregated spots. Moreover, in the LY294002 pretreatment group, the immunostaining of Bad was relatively stronger than that in the ZEN + Gln group. 

### 2.9. Western Blotting

**Figure 9:**
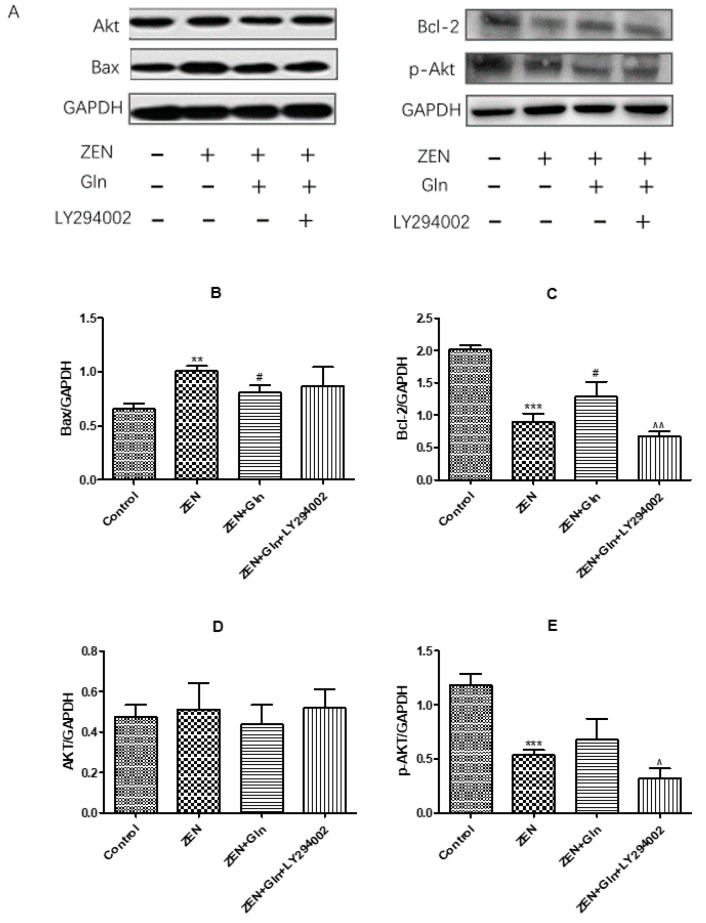
Effects of ZEN, Gln and LY294002 on the PI3K/Akt pathway- and apoptosis-related genes in the IPEC-J2 cells. Values are expressed as means ± SD of three independent experiments. ** *p* < 0.01 and *** *p* < 0.001 ZEN vs. control. # *p* < 0.05 ZEN vs. ZEN + Gln. ^ *p* < 0.05 and ^^ *p* < 0.01 ZEN + Gln vs. ZEN + Gln + LY294002. (**A**) Effects of ZEN, Gln and LY294002 on the protein expression of PI3K/Akt pathway; (**B**) Effects of ZEN, Gln and LY294002 on the protein expression of Bax; (**C**) Effects of ZEN, Gln and LY294002 on the protein expression of Bcl-2; (**D**) Effects of ZEN, Gln and LY294002 on the protein expression of Akt; (**E**) Effects of ZEN, Gln and LY294002 on the protein expression of p-Akt.

To further verify that the PI3K/Akt signaling pathway is the mechanism by which Gln protects against ZEN-induced apoptosis, the related proteins of the PI3K/Akt signaling pathway and apoptosis-related proteins were measured by western blotting. As shown in Figure 9, there was no difference in the protein expression of Akt in the four treatment groups (Figure 9D). Compared with the control group, after ZEN-exposed, the protein expression of pro-apoptotic gene Bax and anti-apoptotic gene Bcl-2 were significantly increased (*p* < 0.01) and significantly decreased (*p* < 0.001), respectively. The addition of Gln significantly decreased the protein expression of Bax compared with the ZEN group (*p* < 0.05), conversely, the protein expression of Bcl-2 was significantly elevated (*p* < 0.05). Compared with the ZEN + Gln group, the pretreatment of LY294002 significantly decreased the protein expression of Bcl-2 (*p* < 0.01), and there was no significant change in Bax protein expression. Also, as shown in Figure 9E, after ZEN-exposed, a remarkable decrease in the protein expression of the p-Akt compared with the control group, the addition of Gln increased the protein expression of p-Akt but did not reach a significant level. In addition, pretreatment with LY294002 significantly reduced p-Akt protein expression.

## 3. Discussion

ZEN is widely found in cereals and animal feed worldwide, which has a negative impact on human and animal health [4,19,20]. Intestinal epithelial cells are the first target of ZEN after ingestion of feed and foods contaminated with ZEN [21]. In vitro and in vivo studies found that oxidative damage was one of the crucial pathways by which ZEN induced cytotoxicity, resulting in cell apoptosis [22,23]. Oxidative damage is mainly caused by the mass production of ROS and free radicals [24]. Oxidative stress is caused by the excessive generation of ROS or the disruption of the oxidoreductase balance in the cell. It not only activated cell signaling but also induced apoptosis [25]. As described above, the results of this study found that ZEN exposure produced excess ROS (Figure 3), numerous uneven nuclear staining, nuclear fissures, mass cells apoptosis (Figure 6). To date, these antioxidant enzymes, comprising CAT, Gpx and GR, etc., are a source of protection against oxidative stress [26,27]. Overall, cells exposed to ZEN induced oxidative damage and reduced the intracellular antioxidant enzyme activities (Figure 2).

Gln, a major substrate utilized by intestinal cells, is not only a source of the main energy of the cell mitochondria, but it can eliminate some of the strong oxidants and protect cells from oxidative damage [28,29]. In gut physiology, the addition of Gln can promote enterocyte proliferation and protect against apoptosis under stress conditions [30]. Therefore, Gln was used to investigate the protective mechanism against ZEN-induced apoptosis in this study. It was observed that the addition of Gln increased cell survival (Figure 1), reduced nuclear shrinkage (Figure 5), and decreased apoptosis rate (Figure 6). The results showed Gln alleviated the apoptosis induced by ZEN. At the same time, the activities of antioxidant enzymes in the cells also increased, and the effect was the best when Gln concentration was 2 mM (Figure 2). Hence, 2 mM Gln was selected as the concentration for subsequent verification. Compared with 2mM Gln, the protective effect of Gln (4 and 8 mM) is weaker. The reason may be consistent with Curi’s conclusion that although Gln supplementation can bring obvious benefits in many cases, the adverse effects of long-term use of high concentrations of glutamic acid might not be completely ruled out [31]. Further, the results of pretreatment with LY294002 that did not reduce nuclear apoptosis are consistent with earlier studies because it has cytostatic, but no cytotoxicity effects on cells [32,33,34].

ZEN exposure can induce IPEC-J2 cells apoptosis by mitochondrial damage [6,7,35]. The literature demonstrated that mitochondria-dependent apoptotic pathways involved a variety of events, such as the production of ROS, the release of Cyto-c in mitochondria, Bcl-2 family members, and activation of caspases-9 and caspases-3 [36]. We found that ZEN induced apoptosis via the mitochondrial pathway of IPEC-J2 cells. The expression of Cyto-c, caspases-9, caspases-3, and pro-apoptotic genes (Bax and Bad) were increased, while anti-apoptotic genes (Bcl-2 and Bcl-xl) were reduced (Figure 7 and Figure 9B,C). In addition, mitochondria are a storage room for intracellular calcium [37]. Recently, with the in-depth discussion of the apoptotic process, it was suggested that intracellular Ca^2+^ and ROS surges were vital mediators of cell death [38,39]. The current study showed that ZEN exposure increased intracellular ROS and Ca^2+^ levels (Figure 3 and Figure 4). Oppositely, the addition of Gln reduced the content of ROS and Ca^2+^ in cells (Figure 3 and Figure 4). These results were consistent with the present study. Overall, Gln improved cell survival rate and protected cells from ZEN-induced mitochondrial apoptosis.

The PI3K/Akt pathway is an important regulator of cellular homeostasis in vivo [40,41]. In addition, it is a vital anti-apoptosis/proliferation signaling pathway that plays a key role in cellular functioning [42,43]. Recently, it was found that Gln increased the antioxidant capacity by activating PI3K/Akt signaling pathway in Parkinson’s disease [44]. Phosphatidylinositol-3 kinases (PI3Ks) are a family of lipid kinases that regulate various metabolic activities in the cell [45]. Activated Akt is a downstream effector of PI3K, which can inhibit apoptosis by regulating multiple targets such as MPTP, ATPase, NF-κB, and the Bcl-2 family proteins [46,47]. We speculated that Gln could exert the effect of anti-apoptosis via PI3K/Akt signaling pathway and improve cell survival. The Bad protein is a downstream substrate of Akt, Akt-phosphorylate (p-Akt) can activate it [48]. In the presence of survival factors, the Bad protein is phosphorylated at two serine sites (Ser-112 and Ser-136) and sequestered in the form of inactive molecules in the cytosol, receiving the death signal, Bad dephosphorylates and interacts with Bcl-xl–Bcl-2 to form dimers that accumulate in mitochondria [49,50]. Therefore, to examine the position of Bad in the case of cell survival and death, immunofluorescence staining was used to observe the fluorescence change of the Bad gene. The results clearly showed that the strong accumulation of Bad gene fluorescence occurred in ZEN-induced cell apoptosis (Figure 8), the mRNA expression of Bad was increased simultaneously. However, there was no accumulation of Bad gene fluorescence occurred, but the mRNA expression of Bad was decreased (Figure 7G), and decreased cell apoptotic rate (Figure 6) when treated with Gln. Importantly, after pretreatment with inhibitor (LY294002) of the PI3K/Akt signaling pathway, the Bad gene strong accumulation of fluorescence occurred (Figure 8). This result suggested that Gln may play an anti-apoptotic effect via the PI3K/Akt signaling pathway. The same result was found in primary liver cancer cells increases of Bad-expressing caused by Akt-knocked [43]. 

The literature suggested that the activation of the PI3K/Akt signaling pathway could suppress apoptosis [46]. Western blotting results in this study showed that Gln increased the expression of p-Akt protein and anti-apoptotic protein Bcl-2 (Figure 9C,E), these results were consistent with the activation of the PI3K/Akt pathway leading to increased expression of Bcl-2 [51]. Our results also showed that Gln treatment stimulated phosphorylation of Akt, and reduced the apoptosis rates (Figure 6), consistent with a previous study showing that growth factor receptor Akt activation prevented apoptosis [52]. Thus, the addition of Gln activated the PI3K/Akt signaling pathway and protected cells from ZEN-induced apoptosis. Meanwhile, pretreatment with LY294002 reduced p-Akt protein expression, suggesting that the anti-apoptotic pathway of PI3K/Akt was inhibited. This result was supported by an early study that LY294002 inhibited the PI3K/Akt signaling pathway and significantly enhanced bufalin-induced apoptosis [53]. As described above, these results suggested that Gln protected cells from ZEN-induced apoptosis, and activation of the PI3K/Akt signaling pathway was one of the factors.

## 4. Conclusions

In conclusion, ZEN exposure induced the excessive generation of ROS, increased intracellular Ca^2+^ concentration, induced oxidative damage, and activated the intrinsic apoptotic cascade reaction in IPEC-J2 cells. However, Gln addition increased the activities of intracellular antioxidant enzymes, increased the expression of anti-apoptotic genes and p-Akt, reduced the expression of pro-apoptotic genes and caspase cascade enzymes. Overall, these findings suggested that Gln antagonized ZEN-induced apoptosis, possibly via the PI3K/Akt signaling pathway in IPEC-J2 cells.

## 5. Materials and Methods

### 5.1. Chemicals and Reagents

The Zearalenone (ZEN) and Glutamine (Gln) were obtained from Sigma-Aldrich (St. Louis, MO, USA) and were dissolved in ethanol and deionized water to a stock solution of 100 mM and 200 mM, respectively. DMEM-F:12 cell culture medium was purchased from Thermo Fisher (Hyclone, Beijing, China). Fetal bovine serum (FBS) was supplied by Gibco-Life Technology (Grand Island, NY, USA). Trypsin/EDTA, Penicillin/streptomycin, the activity assay kits of Catalase (CAT), Glutathione reductase (GR), Glutathione peroxidase (Gpx), and Alkaline phosphatase (AP), ECL detection kit, the Annexin V-FITC/PI apoptosis detection kit, Hoechst-33258 Staining Kit, Reactive Oxygen Species Assay Kit, Fluo-4 AM and BCA Assay Kit were supplied by Beyotime Biotechnology (Nantong, China). Cell Counting Kit-8 (CCK-8) was supplied by Dojindo (Kumamoto, Japan). Phosphate buffered saline (PBS) was purchased from Biotopped (Beijing, China).

### 5.2. Cell and Cell Culture

IPEC-J2 cells were donated by China Agricultural University. The cells were cultured in a complete medium composed of DMEM-F:12 medium (Hyclone, Beijing, China), 10% FBS (GIBCO, Grand Island, NY, USA), and 1% penicillin and streptomycin (Beyotime Biotechnology, Nantong, China). Cells were cultured in an incubator at 37 °C, with a continual supply of 5% CO_2_.

### 5.3. Cell Viability Assay

Based on our previous findings (not yet published), we selected ZEN (160 µM) to infect IPEC-J2 cells. IPEC-J2 cells (0.8–1.0 × 10^5^ cells/mL) were seeded in 96-well culture plates; culture medium was changed every 24 h. When cells became monolayer, cells were washed twice with PBS, then treated with ZEN (160 µM) and different concentrations of Gln (0.5, 1, 2, 4, and 8 mM) for 48 h. IPEC-J2 cells were washed three times with PBS after the cell culture medium was removed, then CCK-8 (10 µL) was added and incubated at 37 °C for 3 h. Cell viability was measured by absorbance on a microplate reader at 450 nm emission wavelength (Tecan Austria GmbH Untersbergatr, Austria). The microplate readers used in this study were from one manufacturer.

### 5.4. Determination of IPEC-J2 Cellular the Activities of Enzymes

IPEC-J2 cells (2.0–2.5 × 10^6^ cells/mL) were grown in 6-well culture plates and treated with drugs as described in the previous section. The CAT, GR, and Gpx activities were determined according to the manufacturer’s instructions. 

### 5.5. Detection of ROS Generation

Changes in intracellular ROS was detected with dichlorofluorescein diacetate (DCFH-DA). IPEC-J2 cells (4.0–5.0 × 10^5^ cells/mL) were grown in 24-well culture plates and treated with drugs as described in the previous section. After treatment, cells were washed thrice with PBS and incubated with 10 µM DCFH-DA at 37 °C for 20 min. Finally, cells were washed thrice with PBS and left a small amount of PBS. Intracellular production of ROS was measured by a microplate reader (Ex = 488 nm and Em = 525 nm). ROS production was expressed as a percentage of the control.

### 5.6. Measurement of Intracellular Calcium (Ca^2+^) Levels 

Changes in intracellular Ca^2+^ were detected by using the intracellular Ca^2+^ indicator Fluo-4 AM. Fluo-4 AM, an acetyl methyl ester derivative of Fluo-4, can penetrate cell membranes. Upon entering the cell, Fluo-4 AM can be cleaved by intracellular esterase to form Fluo-4, which retain in the cell. After treatment, cells were incubated with Fluo-4 AM (1 µM) at 37 °C for 30 min. Intracellular Ca^2+^ was measured by microplate reader (Ex = 488 nm and Em = 520 nm). Ca^2+^ images were obtained using a fluorescence microscope (Life Technologies Crop Bothell, Bothell, WA, USA).

### 5.7. Hoechst-33258 Staining

After treatment, cells were washed 2–3 times with PBS and added 200 µL Hoechst-33258 at room temperature for 3–5 min in the dark. Lastly, aspirated Hoechst-33258 staining solution and washed with PBS 2–3 times, 3–5 min each time. The stained cells were visualized and photographed under a fluorescence microscope (Life Technologies Crop Bothell, Bothell, WA, USA).

### 5.8. Apoptosis Detection

According to the manufacturer’s protocol, the apoptosis rate of IPEC-J2 cells was measured by the Annexin V-FITC/PI apoptosis detection kit. After treatment, the cells were rinsed 2–3 times using PBS, trypsinized, and collected. Next, cells were resuspended in 195 μL of binding buffer. Then, 5 μL of Annexin V- FITC and 10 μL of propidium iodide (PI) were added to the tubes and gently vortexed. Lastly, the cells were analyzed by flow cytometry (Becton, Dickinson and Company, Franklin Lakes, NJ, USA).

### 5.9. Realtime PCR (RT-PCR) Assay

TRIzol reagent (Invitrogen, Shanghai, China) was used to isolate Total RNA from the cells. The concentration of RNA (A260/A280 ratio) was measured by using a Nano Photometer P-Class (IMPLEN, München, Germany). Reverse transcription of 5 μL RNA was performed using the PrimeScript™ RT reagent kit and the concentration of total RNA was 300 ng μL^−1^. SYBR Green I RT-PCR kit (Takara, Dalian, China) was performed in a reaction volume of 10 μL using RT-PCR. Relative quantification of gene expression was calculated using the 2^−ΔΔCt^ method and normalized to GAPDH in each sample. The gene-specific primers are shown in Table 1.

### 5.10. Western Blotting Analyses

The density of each protein was detected by BCA Assay kit. Total protein was loaded onto 6–15% SDS–PAGE gel electrophoresis, separated by electrophoresis, and then was transferred to PVDF membranes. The membranes were blocked with 5% BSA in TBST at room temperature for 2 h, and probed with the indicated primary antibodies: Akt (1:2000, Sangon Biotech, Shanghai, China), p-Akt (1:1000, Cell Signaling Technology, Danvers, MA, USA), Bax, and Bcl-2 (1:1000, Beyotime Institute of Biotechnology, Nantong, China) at 4 °C overnight. Then, the members were washed in TBST three times, incubated with goat anti-rabbit/mouse secondary antibodies (1:1000; Beyotime Institute of Biotechnology, Nantong, China) at room temperature for 2 h and visualized using ECL Plus detection system (P1010, Applygen, Beijing, China). The density of the bands was analyzed using Image J software (National Institutes of Health, Bethesda, Rockville, MD, USA) and normalized to GAPDH.

### 5.11. Immunofluorescence Staining of Cells

IPEC-J2 cells were seeded in a polylysine-coated confocal dish (2.0–2.5 × 10^6^ cells/mL). After treatment, the cells were washed with PBS three times, fixed with 4% polyoxymethylene for 30 min, and 0.2% Triton X-100 for 10 min. Subsequently, 2% BSA-PBS was added dropwise and blocked for 60 min at 37 °C. Cells were stained with primary rabbit anti-Bad (1:1000, Abcam, Cambridge, UK) antibody for one night at 4 °C, followed by incubation with Alexa Fluor 555-conjugated anti-rabbit secondary antibody (1:200; Beyotime Institute of Biotechnology, Nantong, China) for 120 min at 37 °C. Cell nuclei were stained with DAPI (Beyotime Institute of Biotechnology, Nantong, China) for 30 s at room temperature in the dark. 100 µL of PBS was added dropwise and photographed under a fluorescence microscope (Life Technologies Crop Bothell, Bothell, WA, USA).

### 5.12. Statistical Analyses

Data of three independent experiments were expressed as means ± Standard Deviation (SD) and performed using GraphPad Prime 6.0 software (GraphPad Software, Inc, CA, USA). Statistical figures were analyzed using SPSS 19.0 software (SPSS Inc, Chicago, IL, USA). All the experimental data were analyzed for variance uniformity, then analyzed by a one-way ANOVA and groups were compared using LSD’s test. A *p*-value less than 0.05 was considered to indicate statistical significance.

## Figures and Tables

**Table 1:** Primers used for RT-PCR.

Genes	Accession Number	Orientation	Sequence (5′-3′)	Fragments Size (bp)	Tm (°C)
GAPDH	NM_	Forward	GATGGTGAAGGTCGGAGTGAAC	153	60.9
	001206359.1	Reversed	TGGGTGGAATCATACTGGAACA		
Caspase-3	NM_	Forward	GACACTCGCTCAACTTCTTGG	121	54.5
	214131.1	Reversed	TTGGACTGTGGGATTGAGAC		
Caspase-9	XM_	Forward	GGACATTGGTTCTGGAGGATT	116	52.3
	013998997.1	Reversed	TGTTGATGATGAGGCAGTGG		
Cyto-c	NM_	Forward	CTCTTACACAGATGCCAACAA	139	56.1
	001129970.1	Reversed	TTCCCTTTCTCCCTTCTTCT		
Bax	XM_	Forward	TTTGCTTCAGGGTTTCATCC	113	54.4
	003127290.3	Reversed	GACACTCGCTCAACTTCTTGG		
Bcl-2	AB	Forward	GCGACTTTGCCGAGATGT	116	55.9
	271960.1	Reversed	CACAATCCTCCCCCAGTTC		
Bcl-xl	XM_	Forward	GCAGGTAGTGAACGAACTCTTCCG	140	60.08
	021077298.1	Reversed	CCATCCAAGTTGCGATCCGACTC		
Bad	XM_	Forward	CTTACCCAGAGGGGACCGAG	153	58.39
	021082883.1	Reversed	AGGAACCCTGGAACTCGTCA		

## Data Availability

The data presented in this study are openly available in this article.
